# South East Asian Society of Interventional Radiology (SEASIR): state and future of radiology in interventional radiology

**DOI:** 10.2349/biij.5.4.e26

**Published:** 2009-10-01

**Authors:** BJJ Abdullah

**Affiliations:** Department of Biomedical Imaging, University of Malaya, Kuala Lumpur, Malaysia

The empires of the future are the empires of the mind

-Sir Winston Churchill

Healthcare is widely viewed as an essential service and even a right of all members of society. Global dynamic changes within the past decade in the legislative, financial and technological areas have had a dramatic effect on the practice of radiology. Interventional radiology is no exception. The provision of basic healthcare with escalating costs of healthcare, aging populations, global financial crises, higher expectations of the citizens, imminent introduction of the ASEAN Free Trade Area (AFTA) with its Roadmap for Integration of the healthcare sector [1], health tourism as well as the increased use of teleradiology are some of the numerous challenges facing radiological practice in the ASEAN region. Further, administrative changes within the healthcare sector are increasingly moving power to the administrators.

Interventional radiology is a clinical discipline with a procedural foundation rooted in diagnostic imaging and almost entirely dependent on innovation. Like its diagnostic “sibling”, interventional radiology has a very strong clinical focus that demands a “patient-centred” focus. Interventional radiologists possess a special blend of knowledge based on diagnostic imaging fundamentals complemented by technical and clinical management expertise that, when applied skillfully and with care, can save and improve lives cost-effectively. The advent of more robust and user-friendly devices with decreased cost is making procedures available to a greater number of patients. Further, the emergence of techniques such MR guided Focused Ultrasound Surgery (MRgFUS) will create a new breed of “computer game” image-guided interventionalists.

Today the number of interventional radiology (IR) procedures continues to grow at a rapid pace with newer techniques based on rapid advances in technology. Unfortunately, there is a concomitant shortage of expertise which is also affecting the diagnostic radiology. This shortage is expected to worsen, with some estimating a 2% annual increase in the number of radiologists, compared with a 3.5% increase in the number of procedures. The compensation structures which favour diagnostic procedures over interventional procedures (along with the greater risks associated with performing interventional procuress) has drawn away young interventional radiologists into the diagnostic side. Interestingly, even though there is recognition of the importance on teaching diagnostic radiology to medical students, relatively little has been done to attract medical students to radiology, especially interventional radiology. Additionally the political influence of interventional radiologists is visibly lacking and it is imperative that interventional radiologists gain recognition as substantial contributors to the advancement of medicine at the level of government funding agencies for the specialty to grow.

Interventional radiology, which is still young in the ASEAN region, has made tremendous progress over the last 2 decades. It has to be said that this progress has occurred in varying degrees with good interventional services in some regions or countries (e.g. Singapore and Thailand) and virtually non-existent in others. There are only a handful of interventional radiologists who are practising interventional radiology and most only on a part-time basis. This is related mainly to the lack of resources and poorly developed referral patterns secondary to issues of turf. Credit must go to the earlier pioneers who recognised the tremendous potential of the new discipline and made a concerted effort to ensure interventional radiology’s rightful place in the community of medicine in their respective countries. They faced tough challenges, among them a lack of support from colleagues, lack of adequate numbers of radiologists, and lack of financial support. Despite the odds they were able to create the vision, excite the younger colleagues and emphasise the importance of the future role of interventional radiology. This early success was built upon by the current leaders within each of the ASEAN countries who are also working tirelessly to sustain the future of interventional radiology.

The SEASIR was formed to overcome some of these challenges and the stated objectives of the SEASIR are:

1. To advance interventional radiology through a non-profit society of radiologists and individuals in related fields of medicine and science in South East Asia.
2. To encourage and support the development of expertise and training in interventional radiology in South East Asia.
3. To foster the promotion of closer fellowship, collaboration and exchange of ideas among individuals with an interest in interventional radiology and related fields in South East Asia.
4. To provide scientific meetings for the reading and discussion of papers, research, preliminary work and ideas, and the dissemination of knowledge related to interventional radiology.
5. In furtherance of the above objects, the Society may provide continuing education through refresher courses, and disseminate knowledge through publications of the Society.

Member countries to date consist of Thailand, Indonesia, Singapore, Malaysia, Vietnam and Philippines. Over the last several years the network amongst the interventional radiologists in SEASIR has grown and strengthened with a regular exchange of speakers and trainers to share and develop skills amongst its member nations.

SEASIR was formed following a meeting held in Bali, Indonesia on 7th Sept 2002. Dr Prijo Sidipratomo from Indonesia was the founding president. To date six business meetings have been held. We are currently developing a modest website hosted by the Thailand Society of Interventional Radiology [2]. There is still a long way for interventional radiology in the SEA region to reach the levels of its bigger and older regional siblings e.g. Korea, Japan and India. Hopefully from these modest beginnings, we will be able to lay a good foundation for our future fellow interventional radiologists to grow this specialty and bring it to its rightful place, which is among the leaders in medical practice in the region.

Amidst this complex and challenging background, this special issue aims to recognise those early pioneers, chart the course of interventional radiology, view the current status, and explore the future direction and potential challenges facing interventional radiology within the different countries of ASEAN. This sharing of experiences and approaches would assist others to learn from our experiences so that the practice of interventional radiology can be enhanced for the betterment of the communities we work and live in.

We take this opportunity to thank the leaders in interventional radiology who have driven us thus far and the contributors who have made this issue a success.
